# Dynamics and implications of circulating anti-angiogenic VEGF-A_165_b isoform in patients with ST-elevation myocardial infarction

**DOI:** 10.1038/s41598-017-10505-9

**Published:** 2017-08-30

**Authors:** Luisa Hueso, Cesar Rios-Navarro, Amparo Ruiz-Sauri, Francisco Javier Chorro, Julio Nunez, Maria Jesus Sanz, Vicente Bodi, Laura Piqueras

**Affiliations:** 1 Institute of Health Research-INCLIVA, Valencia, Spain; 2 Cardiology Department, Hospital Clinico Universitario, Valencia, Spain; 3 Pathology Department, Faculty of Medicine, University of Valencia, Valencia, Spain; 4 Medicine Department, Faculty of Medicine, University of Valencia, Valencia, Spain; 5 Centro de Investigación Biomédica en Red - Cardiovascular (CIBER-CV), Madrid, Spain; 6 Pharmacology Department, Faculty of Medicine, University of Valencia, Valencia, Spain

## Abstract

Angiogenesis is crucial to restore microvascular perfusion in the jeopardized myocardium in the weeks following reperfused ST-segment elevation myocardial infarction (STEMI). (VEGF)-A_165_b, an anti-angiogenic factor, has been identified as a regulator of vascularization; however, it has not been previously implicated in acute myocardial infarction. We sought to investigate the dynamics of circulating VEGF-A_165_b and its association with cardiac magnetic resonance-derived infarct size and left ventricular ejection fraction (LVEF). 50 STEMI patients and 23 controls were included. Compared with control individuals, serum VEGF-A_165_b was elevated in STEMI patients prior to primary percutaneous coronary intervention (PCI). Following PCI, serum VEGF-A_165_b increased further, reaching a maximum level at 24 h and decreased one month after reperfusion. VEGF-A_165_b levels at 24 h were associated with a large infarct size and inversely related to LVEF. VEGF-A_165_b expression was increased in myocardial infarct areas from patients with previous history of AMI. An *ex vivo* assay using serum from STEMI patients showed that neutralization of VEGF-A_165_b increased tubulogenesis. Overall, the study suggests that VEGF-A_165_b might play a deleterious role after AMI as an inhibitor of angiogenesis in the myocardium. Accordingly, neutralization of VEGF-A_165_b could represent a novel pro-angiogenic therapy for reperfusion of myocardium in STEMI.

## Introduction

Coronary artery disease (CAD) is the most common cause of mortality worldwide. The most prevalent manifestation of CAD is acute myocardial infarction (AMI), which is characterized by myocardial damage due to prolonged ischemia. Even in promptly reperfused ST-segment elevation myocardial infarction (STEMI), severe microvascular damage occurs in a significant number of patients and associates with more extreme left ventricular (LV) structural deterioration, worsening patient outcomes^1, 2^. Angiogenesis plays a critical role in myocardial repair in the days and weeks following AMI^3^. Impaired neovascularization in the infarct area and the resultant metabolic imbalance are important contributors to the transition to heart failure, the main cause of death in AMI patients in a long-term perspective^3^.

Vascular endothelial growth factor-A (VEGF-A) plays a crucial role not only in physiological angiogenesis, but also in pathological angiogenesis^3–5^. VEGF-A is markedly increased in the ischemic myocardium^6, 7^, and elevated VEGF-A serum levels have been reported after AMI in patients^8, 9^. However, despite the high levels of VEGF-A after acute ischemia, neovascularization remains insufficient in the infarcted area in a significant number of patients.

VEGF-A is generated as multiple mRNA isoforms through alternative splicing, which includes a proximal splice-site selection in exon 8 to produce an exon 8a sequence, resulting in the pro-angiogenic VEGF-A_165_, while distal splice-site selection generates exon 8b, yielding the anti-angiogenic isoform VEGF-A_165_b^10^. VEGF-A_165_b exhibits similar binding affinity as VEGF-A_165_ to vascular endothelial growth factor receptor-2 (VEGFR-2) but fails to activate receptor phosphorylation, consequently impairing angiogenesis^11, 12^. In humans, downregulation of VEGF-A_165_b has been documented in a large number of angiogenic pathological states such as proliferative diabetic retinopathy^13^ and cancer^12, 14–16^. Conversely, increased levels of VEGF-A_165_b have been associated with impaired vascularization in patients with peripheral artery disease^17–19^ and systemic sclerosis^20^. However, there have been no reports examining the VEGF-A_165_b isoform in AMI.

In the present study, we hypothesized that circulating levels of VEGF-A_165_b are altered after AMI and might reflect the extent of LV damage. Additionally, we hypothesized that functional blockade of VEGF-A_165_b in STEMI could boost angiogenesis.

Therefore, the objectives of this study were the following: 1) to evaluate in a prospective series of STEMI patients managed according to current recommendations, the temporal changes in circulating VEGF-A_165_b, and 2) its association with the presence of extensive LV damage as derived from the two most validated indices (LV ejection fraction [LVEF] and infarct size) using cardiac magnetic resonance (CMR); 3) to determine whether VEGF-A_165_b protein expression can be detected in human heart tissue of patients with previous history of AMI; and 4) to investigate the effects of VEGF-A_165_b blockade on angiogenesis using serum from STEMI patients in *ex vivo* assays.

## Results

**Figure 1:**
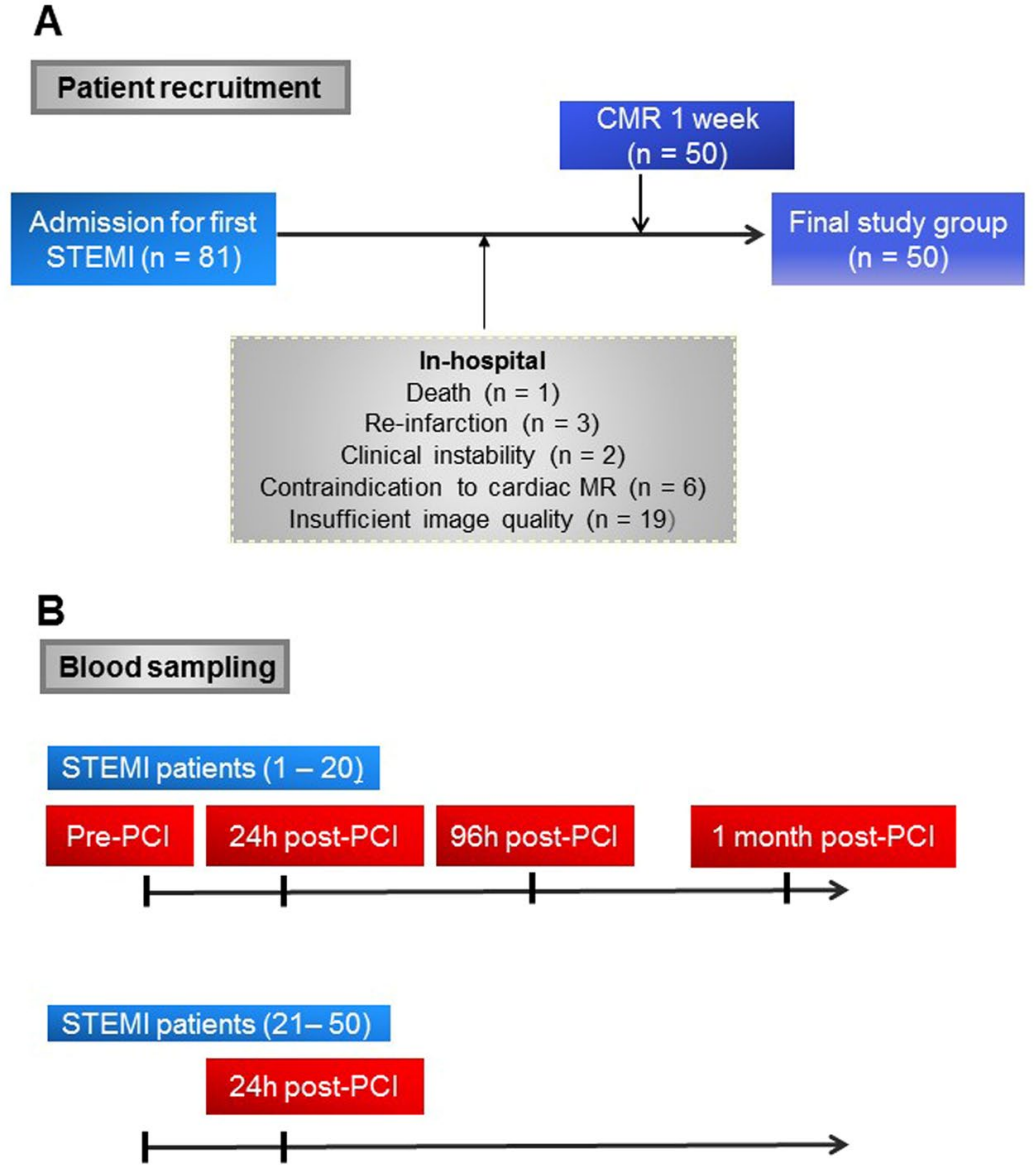
Flow chart showing the enrollment protocol of STEMI patients (**A**) and blood sampling (**B**). PCI, percutaneous coronary intervention; CMR, cardiac magnetic resonance; STEMI, ST-segment elevation myocardial infarction.

Blood samples were obtained from 50 STEMI patients and 23 control subjects. Data regarding patients’ recruitment and blood sampling are shown in Fig. 1A and B, respectively. The characteristics of STEMI patients are shown in Table 1.

**Table 1:** Baseline characteristics, therapies at discharge and CMR characteristics of STEMI patients.

Characteristics (mean ± SD)	STEMI (n = 50)
Age (years)	59 ± 12
Male sex, n (%)	36 (72)
Diabetes mellitus, n (%)	8 (16)
Hypertension, n (%)	22 (44)
Hypercholesterolemia, n (%)	22 (44)
Smoker, n (%)	32 (64)
BMI (kg/m^2^)	27 ± 4
Creatinine (mg/dL)	0.9 ± 0.2
GFR (mL/min/1.73 m^2^)	85 ± 20
PAD, n (%)	1 (2)
Heart rate (beats per minute)	82 ± 19
Systolic blood pressure (mmHg)	125 ± 26
Killip class, n (%)	
I	44 (88)
II	4 (8)
III	1 (2)
IV	1 (2)
Time to reperfusion (min)	210 [128–270]
ST-segment resolution (%)	79 ± 29
Anterior infarction, n (%)	27 (54)
Multivessel disease, n (%)	6 (12)
TIMI flow grade before PCI, n (%)	
0	23 (46)
1	2 (4)
2	5 (10)
3	20 (40)
TIMI flow grade after PCI, n (%)	
0	1 (2)
1	1 (2)
2	6 (12)
3	42 (84)
**Medication**	
Aspirin, n (%)	30 (60)
Clopidogrel, n (%)	2 (4)
Beta-blockers, n (%)	15 (30)
ACE/AR inhibitors, n (%)	17 (34)
Statins, n (%)	25 (50)
Diuretics, n (%)	4 (8)
**CMR data**	
LVEF, %	51 ± 12
LV end-systolic volume index (mL/m^2^)	40 ± 18
LV end-diastolic volume index (mL/m^2^)	79 ± 22
LV mass (g/m^2^)	73 ± 16
Edema (% of LV mass)	30 ± 16
Microvascular obstruction (% of LV mass)	0 [0–2]
Infarct size (% of LV mass)	19 ± 15
Myocardial salvage index (%)	27 [16–69]

Values represent mean ± SD or the percentage of patients. PCI, percutaneous coronary intervention; LVEF, left ventricular ejection fraction; CMR, cardiac magnetic resonance; TIMI, thrombolysis in myocardial infarction; ACE, angiotensin converting enzyme; AR, angiotensin receptor; BMI, body mass index; GFR, glomerular filtration rate; PAD, peripheral artery disease.

### VEGF-A_165_b levels are increased in the circulation of STEMI patients

**Figure 2:**
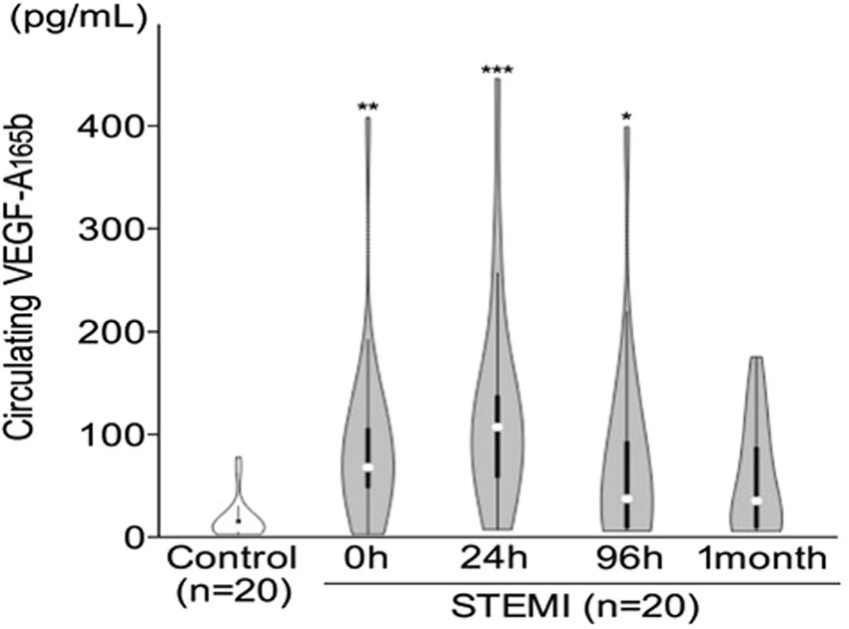
Temporal changes in levels of serum VEGF-A_165_b in STEMI patients and control subjects. Data are from controls (n = 20) and STEMI patients (n = 20) before reperfusion (time = 0 h) and at 24 h, 96 h and 1 month after primary percutaneous coronary intervention. Violin plots shows median values, interquartile range and 95% confidence intervals. ***p < 0.001, **p < 0.01, *p < 0.05 vs control subjects.

To investigate the temporal changes in serum VEGF-A_165_b levels, we performed repeated measurements at defined intervals in the first twenty STEMI patients and twenty controls included in our final study group. The serum concentration of VEGF-A_165_b upon patients’ arrival (before percutaneous coronary intervention [PCI], 0 h) and at 24 h, 96 h and 1 month after reperfusion therapy are shown in Fig. 2. Circulating VEGF-A_165_b levels, even before reperfusion, were significantly higher in STEMI patients (median 64.46 pg/mL, range 0 to 401.3 pg/mL, p < 0.01) than in controls (median 6.36 pg/mL, range 0 to 74.4 pg/mL). VEGF-A_165_b levels peaked 24 h after reperfusion (median 103.3, range 4.5 to 438.9 pg/mL) (p < 0.001) and remained significantly elevated at 96 h after reperfusion (median 34.2 pg/mL, range 2.9 to 392.5 pg/mL, p < 0.05); however, levels decreased after 1 month in STEMI patients (32.2 pg/mL, range 2.7 to 171.1 pg/mL) and were not significantly different to those of control subjects (p > 0.05) (Fig. 2).

### VEGF-A_165_b levels are elevated in STEMI patients with large infarct size and reduced LVEF

**Figure 3:**
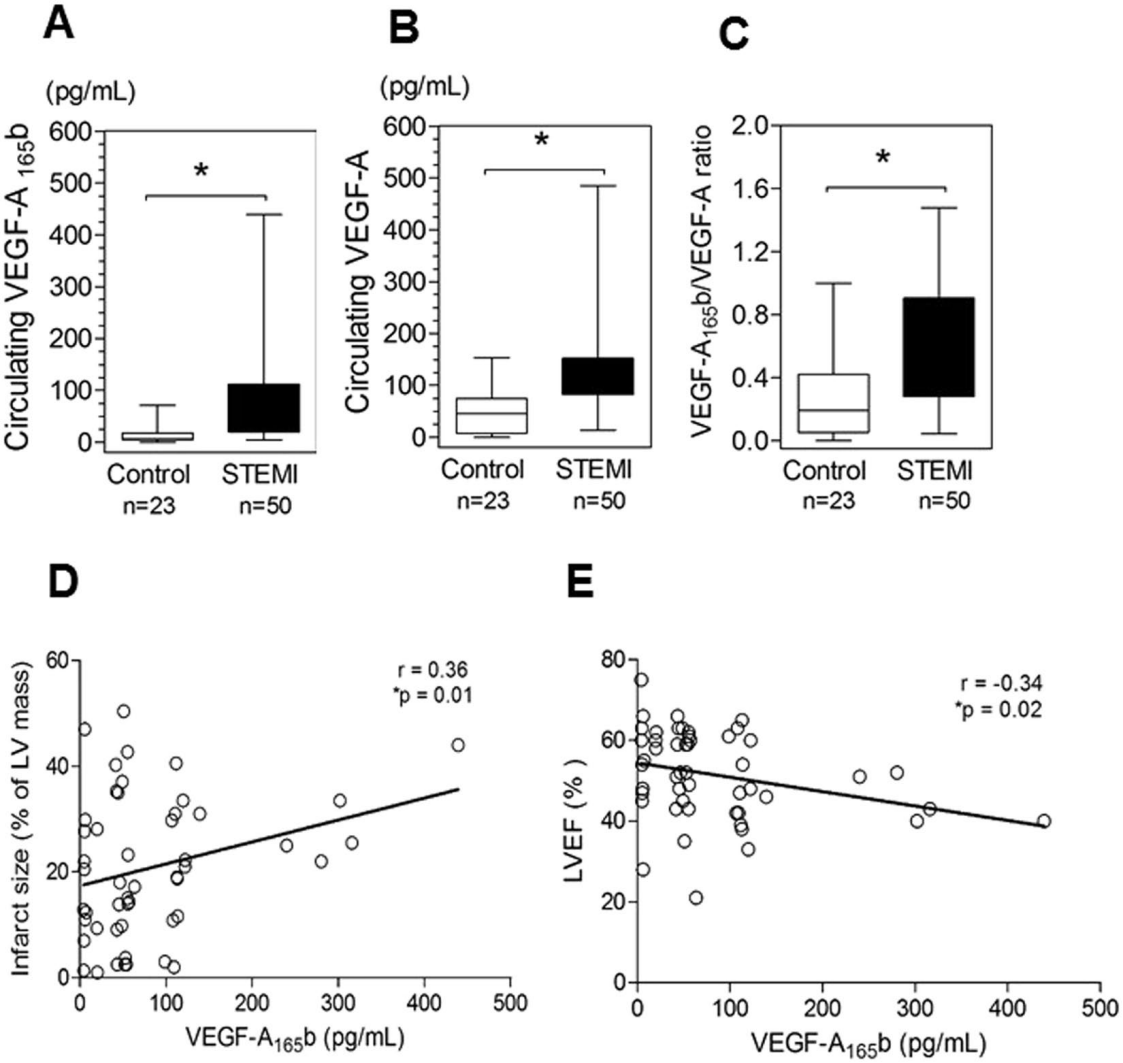
VEGF-A_165_b levels in STEMI patients are associated with larger infarct size and reduced left ventricular ejection fraction (LVEF). (**A**) Circulating VEGF-A_165_b levels (**B**) total VEGF-A levels and (**C**) VEGF-A_165_b/VEGF-A ratio in STEMI patients (n = 50) at 24 h after reperfusion and in control subjects (n = 23). Box plots show median and range values. *p < 0.05. (**D**) Spearman test correlations between VEGF-A_165_b and infarct size and (**E**) LVEF in STEMI patients.

Once the time course of VEGF-A_165_b was defined in the first 20 STEMI patients, and taking into account that VEGF-A_165_b levels peaked 24 h after reperfusion, we sought to evaluate the relationship between VEGF-A_165_b at this time point and the presence of structural damage in pre-discharge CMR. To do this, we extended the study group to 23 controls and recruited 30 additional STEMI patients in whom blood samples were obtained at 24 h. Once again, in the whole study group, serum VEGF-A_165_b levels at 24 h were significantly higher in STEMI patients (n = 50) than in controls (n = 23) (p < 0.01) (Fig. 3A). Additionally, we measured total VEGF-A in both groups. VEGF-A levels were significantly higher in STEMI patients (median 110.4 pg/mL, range 13.9 to 485.3 pg/mL, p < 0.01) than in controls (median 45.83 pg/mL, range 0 to 153.7 pg/mL) (Fig. 3B). When we evaluated the ratio of VEGF-A_165_b/VEGF-A, we observed a significant 2-fold increase in VEGF-A_165_b in STEMI *versus* controls (Fig. 3C, p < 0.05), indicating that total VEGF-A includes □ 60% VEGF-A_165_b fraction in STEMI patients. Moreover, there was a significant association between VEGF-A_165_b serum levels and infarct size (r = 0.36, p = 0.01, Fig. 3D) and an inverse correlation with LVEF (r = −0.34, p = 0.02, Fig. 3E).

### VEGF-A_165_b is upregulated in the infarct areas in patients with a previous history of AMI

Immunohistochemical studies were performed to assess the expression of VEGF-A_165_b in human heart samples. Myocardial sections from four patients with old myocardial infarction (OMI) and five control subjects were immunostained with a mouse monoclonal antibody that specifically detects the VEGF-A_165_b splice variant^20^. The baseline characteristics and autopsy results of patients are described in Table 2. History of AMI had been previously documented in all patients and autopsies unequivocally revealed fibrotic scar tissue characteristic of chronic infarct in all cases. Previous history of ischemic or any other cardiac diseases in controls had been ruled out both in the clinical records and in the autopsy analyses.

**Table 2:** Clinical data and autopsy results of patients.

	Clinical Data	Autopsy Results
Patient 1	77-year-old male	Infarct scar area: 2 × 3 cm lateral wall of the left ventricle
	Time elapsed since infarction: 7 years	Infarct scar area: 2.5 × 0.7 cm interventricular septum
	Cause of death: heart failure	
Patient 2	78-year-old male	Infarct scar area: interventricular septum^a^
	Time elapsed since infarction: 1 year	
	Cause of death: cardiogenic shock	
Patient 3	55-year-old male	Infarct scar area: 2 × 2 cm interventricular septum
	Time elapsed since infarction: 1 year	
	Cause of death: stroke	
Patient 4	58-year-old male	Infarct scar area: 6.5 × 7.5 cm anterior wall of the right ventricle
	Time elapsed since infarction: 1 year	
	Cause of death: reinfarct	

^a^In patient 2, infarct size was not quantified, but was visually described in the autopsy report.

**Figure 4:**
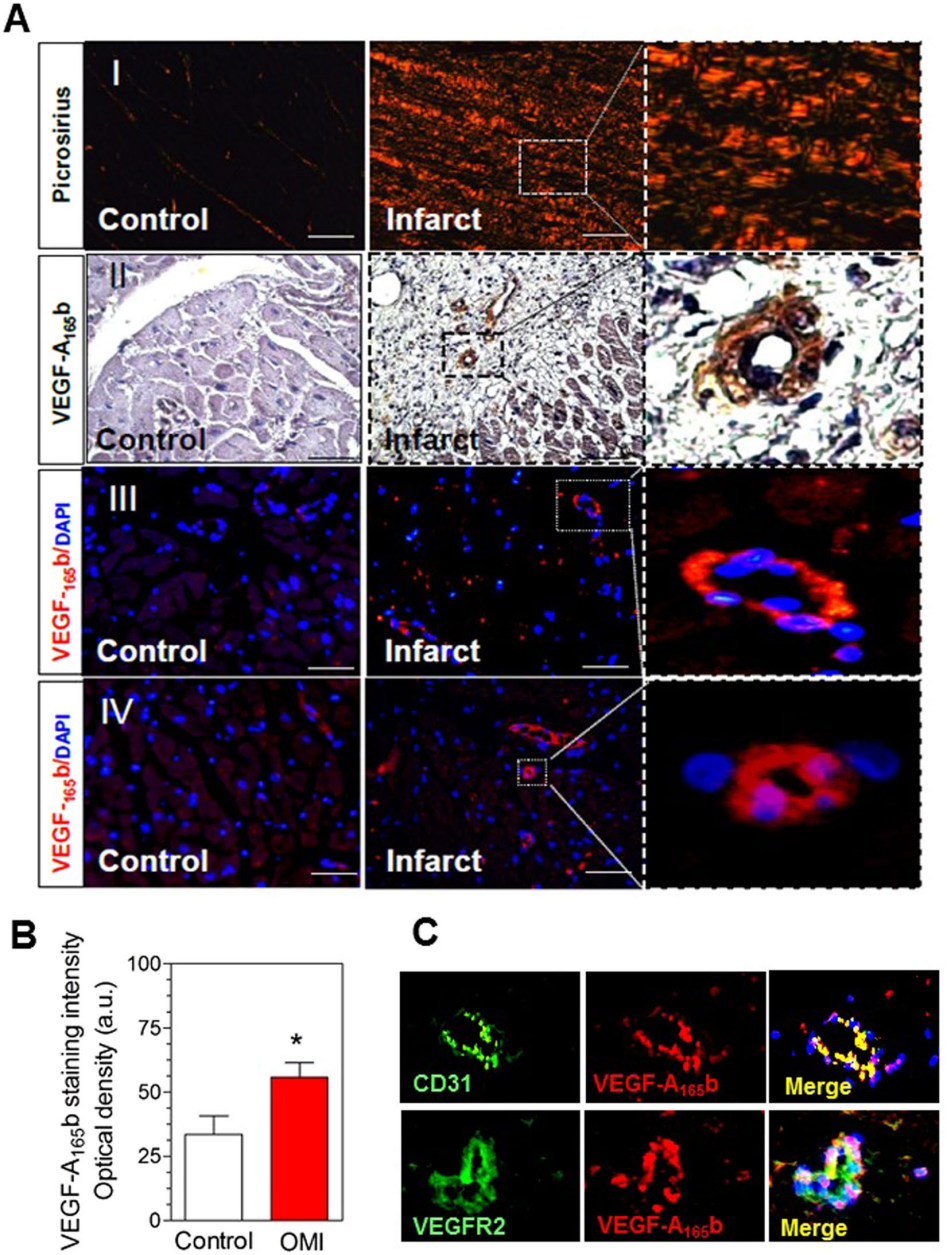
Immunohistochemistry analysis of VEGF-A_165_b expression in heart tissue from autopsies of patients with previous history of myocardial infarction. (**A**) (I) representative images of picrosirius staining. (II) Myocardial sections were incubated with a mouse anti-human VEGF-A_165_b antibody (5 μg/ml) and specific labeling was detected with a biotin-conjugated goat anti-mouse secondary antibody. (III) and (IV) Myocardial sections were incubated with a mouse anti-human VEGF-A_165_b antibody (5 μg/ml) and immunoreactivity was visualized using Alexa Fluor 594 (VEGF-A_165_b, *red*) secondary antibodies. Nuclei were stained with DAPI (*blue*). Bars = 500 μm. (**B**) Densitometric analysis of VEGF-A_165_b immunofluorescent staining. Images from infarct and control sections were captured and digitized (Axio Observer A1, Carl Zeiss) and then analyzed with Image-Pro Plus analysis software (Media Cybernetics). Scoring was performed blinded on coded slides. Data represent mean ± SD of optical density in arbitrary units (a.u.). *p < 0.05 vs control. (**C**) Representative images showing colocalization of CD31/VEGF-A_165_b or VEGFR-2/VEGF-A_165_b in infarct myocardial tissue. Immunoreactivity was visualized using Alexa Fluor 488 (CD31 or VEGFR2, *green*) and Alexa Fluor 594 (VEGF-A_165_b, *red*) secondary antibodies. Nuclei were stained with DAPI (*blue*).

Consistent with our previous findings^21^, histological evaluation of heart sections by picrosirius staining showed myocardial fibrosis and extensive collagen deposition within the infarcted areas (Fig. 4A). Of interest, whereas a weak constitutive expression of VEGF-A_165_b was observed mainly in endothelial cells in myocardial sections from controls, a marked upregulation of VEGF-A_165_b endothelial expression was observed in infarct samples from AMI patients (Fig. 4A). Quantification of immunostaining revealed that VEGF-A_165_b expression was significantly higher in infarcted myocardial sections than in control sections (Fig. 4B, p < 0.05). Furthermore, double labeling immunofluorescence using an endothelial antibody confirmed the expression of VEGF-A_165_b in endothelial cells (CD31+) (Fig. 4C). Additionally, coexpression of VEGF-A_165_b and VEGFR-2 was detected in infarcted myocardium regions (Fig. 4C).

### Neutralization of VEGF-A_165_b activity in serum from STEMI patients increases endothelial network formation

**Figure 5:**
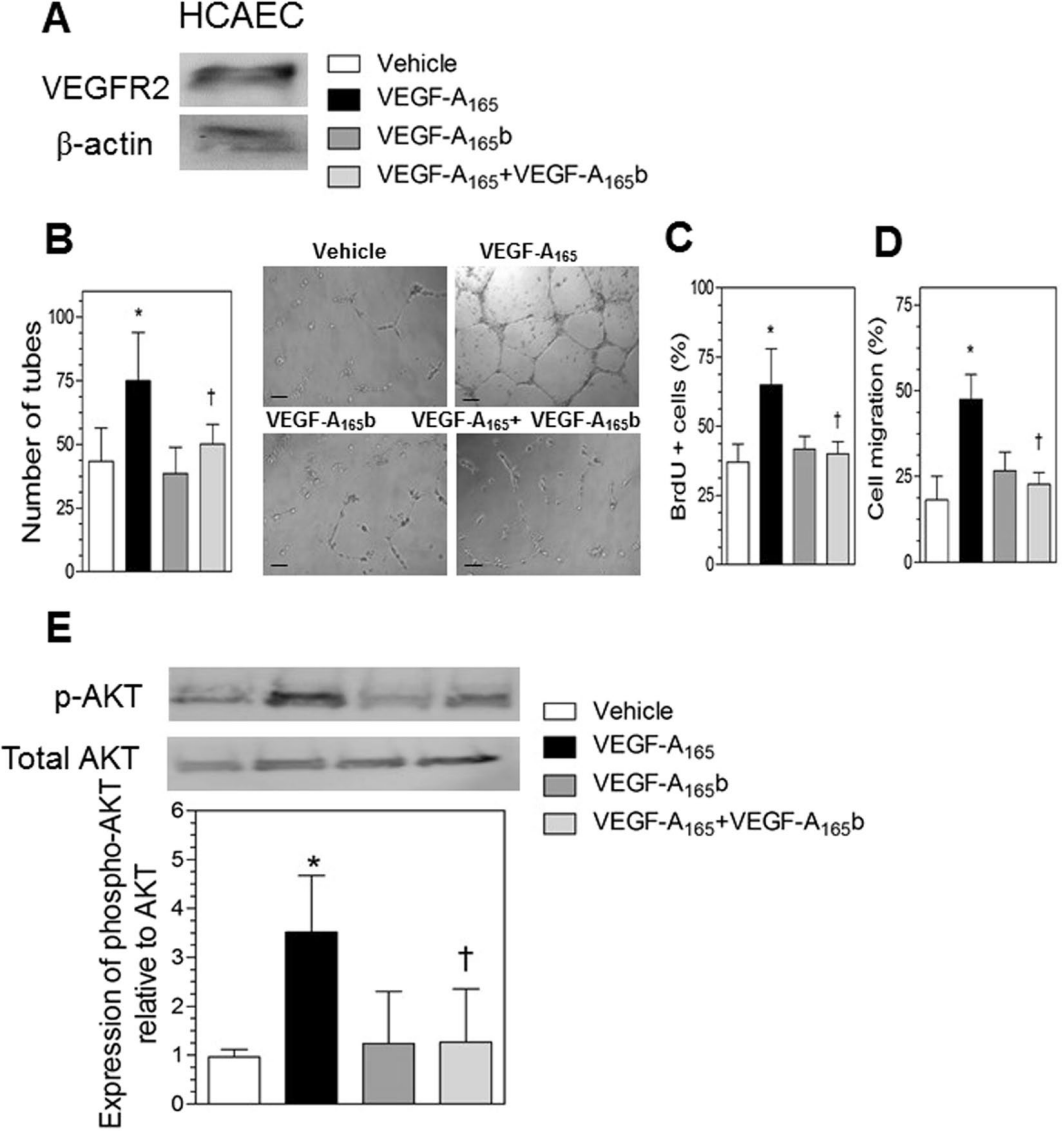
VEGF-A_165_b inhibits VEGF-A_165_-induced angiogenesis of human coronary artery endothelial cells (HCAEC). (**A**) Western blotting of VEGFR2 expression in HCAEC. HCAEC were treated with vehicle (PBS), human recombinant VEGF-A_165_ (30 ng/mL), recombinant VEGF-A_165_b (30 ng/mL) or their combination. (**B**) The number of tube-like structures was determined after 24 h. Data represent the mean ± SD of the number of tubes in 5 low-magnification (×100) fields (n = 6 independent experiments performed in triplicate). *p < 0.01 vs vehicle; ^†^p < 0.05 vs VEGF-A_165_-treated cells. Right panels show representative images of endothelial cell differentiation on Matrigel. Bars = 300 μm. (**C**) Effect of VEGF-A_165_b on proliferation. Results are expressed as percentage of proliferating endothelial cells analyzed by BrdU incorporation. Data represent the mean ± SD (n = 5 independent experiments). *p < 0.01 vs vehicle; ^†^p < 0.05 vs VEGF-A_165_-treated cells. (**D**) Effect of VEGF-A_165_b on migration. Results are expressed as percentage of cell migration analyzed by the wound healing assay. Data represent the mean ± SD. (n = 4 independent experiments) *p < 0.01 vs vehicle; ^†^p < 0.05 vs VEGF-A_165_-treated cells. (**E**) Representative western blots of phospho AKT/total AKT in HCAEC treated with vehicle, human recombinant VEGF-A_165_ (30 ng/mL), recombinant VEGF-A_165_b (30 ng/mL) or their combination for 20 min. Data represent the mean±SD of protein densitometry (n = 3). *p < 0.05 vs vehicle-treated cells ^†^p<0.05 vs VEGF-A_165_-treated cells.

Previous studies have shown that VEGF-A_165_b binds to VEGFR-2 and inhibits angiogenesis elicited by proagiogenic VEGF-A_165_ in several types of endothelial cells^18, 20, 22–25^. However, its effect on human coronary artery endothelial cells (HCAEC) remains unknown. We thus carried out an *in vitro* endothelial differentiation assay^26, 27^ to assess the impact of recombinant VEGF-A_165_b on HCAEC differentiation into capillary-like structures. We first confirmed that VEGFR-2 protein was expressed in HCAEC by western blotting (Fig. 5A). We found that the ability of HCAEC to form tubular-like structures was markedly enhanced in the presence of the angiogenic human recombinant VEGF-A_165_ isoform for 24 h (74.9±19.0 vs control 43.3±13.0, p < 0.01, Fig. 5B). Of note, whereas treatment of HCAEC with VEGF-A_165_b alone did not impact angiogenesis, cotreatment of HCAEC with VEGF-A_165_b significantly reduced the angiogenic response triggered by VEGF-A_165_ (p < 0.05, Fig. 5B).

Given that proliferation and migration of endothelial cells are essential steps in angiogenesis, we also studied the effect of VEGF-A_165_b on these parameters. In the proliferation assay, we observed that treatment of HCAEC with human recombinant VEGF-A_165_ significantly increased the proliferation of HCAEC (64.9±13.0% vs 37.3±6.5% in controls, p < 0.05, Fig. 5C and Supplementary Fig S1). VEGF-A_165_b alone did not impact HCAEC proliferation, however, it significantly decreased VEGF-A_165_-induced HCAEC proliferation (Fig. 5C and Supplementary Fig S1, p < 0.05). Additionally, we measured the effect of VEGF-A_165_b on VEGF-A_165_-mediated migration. Migration was significantly greater in VEGF-A_165_-treated endothelial cells than in vehicle-treated cells (47.9±15.9% vs 18.1±1.5% in controls, p < 0.05, Fig. 5D and Supplementary Fig S1). Whereas VEGF-A_165_b treatment alone did not impact migration, a significant reduction in the migratory potential of VEGF-A_165_-stimulated HCAEC was observed when cells were cotreated with VEGF-A_165_ plus VEGF-A_165_b (Fig. 5D, p < 0.05).

VEGF-A_165_ has been previously shown to stimulate AKT phosphorylation in endothelial cells *via* VEGFR-2 activation^28^. To determine whether VEGF-A_165_b modulates VEGF-A_165_-mediated signaling in human coronary endothelial cells, we treated HCAEC with vehicle, VEGF-A_165_, VEGF-A_165_b, or with both isoforms for 20 min, and then extracted proteins for western blotting. We found that AKT phosphorylation was significantly higher in HCAEC treated with VEGF-A_165_ than in cells treated with vehicle (Fig. 5E). Whereas treatment with VEGF-A_165_b alone did not affect AKT phosphorylation in HCAEC, VEGF-A_165_b significantly reduced VEGF-A_165_-mediated phosphorylation of AKT (p < 0.05, Fig. 5E).

**Figure 6:**
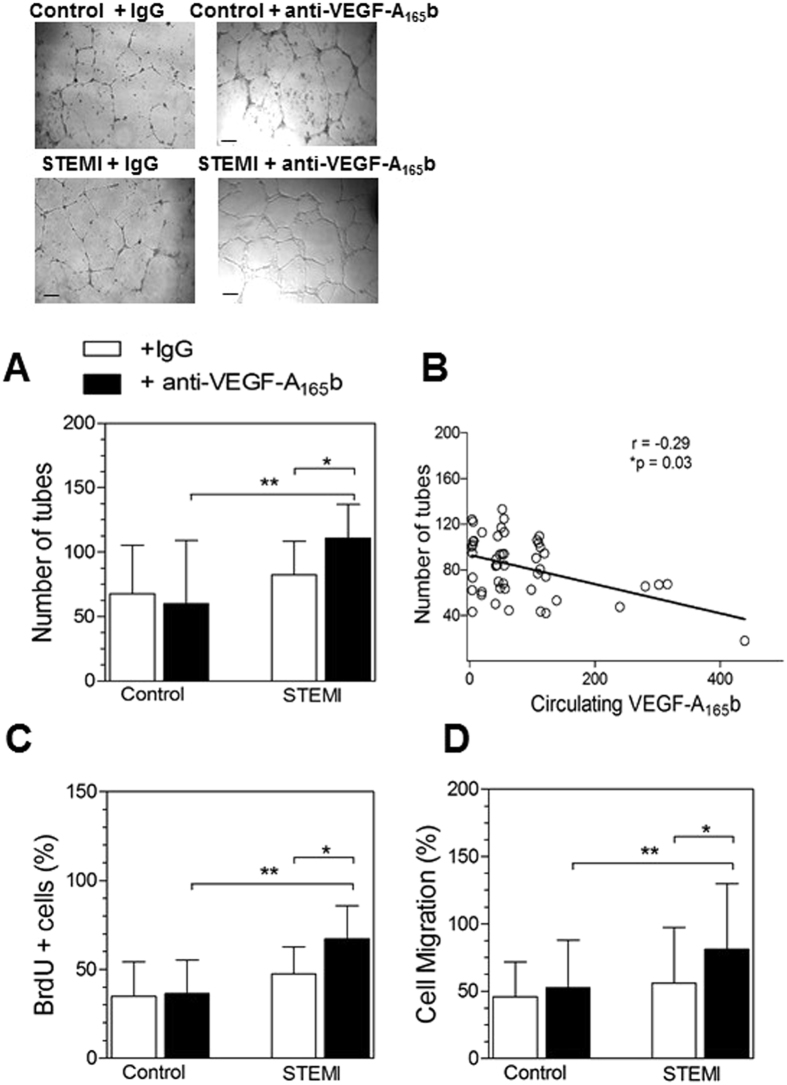
Blocking serum VEGF-A_165_b from STEMI patients with a neutralizing antibody induces angiogenesis. HCAEC were incubated with diluted serum (10%) from STEMI patients (n = 50) or controls (n = 23). Samples were incubated in the presence of a mouse monoclonal anti-human VEGF-A_165_b blocking antibody (10 µg/mL) or irrelevant isotype- and concentration-matched IgG. (**A**) Phase contrast images were taken after 24 h and the number of tube-like structures was counted. Data represent mean ± SD of the number of tube-like structures in 5 low-magnification (×100) fields. Bars = 300 μm. Upper panels show representative images of endothelial cell differentiation on Matrigel. *p < 0.05 **p < 0.01. (**B**) Correlation between VEGF-A_165_b levels and the number of tubes induced by serum (+IgG control) from STEMI patients. (**C**) Percentage of proliferating endothelial cells were analyzed by BrdU incorporation. Data represent the mean ± SD. *p < 0.05 **p < 0.01. (**D**) Endothelial cell migration (%) was analyzed by a wound healing assay. Data represent the mean ± SD. *p < 0.05 **p < 0.01.

Given our findings of increased circulating levels of VEGF-A_165_b in STEMI patients, we further explored the consequences of functional VEGF-A_165_b serum neutralization for angiogenesis *ex vivo*. In the presence of an irrelevant antibody, serum from STEMI patients showed a similar HCAEC angiogenic response to that of the control group (STEMI: 82.6±26.0 vs control: 67.8±37.7, p > 0.05) (Fig. 6A). The addition of anti-VEGF-A_165_b neutralizing antibody, however, resulted in a significant stimulation of tubulogenesis by serum from STEMI patients as compared with control serum (STEMI: 110.7±26.3 vs control: 60.1±49.0, p < 0.01 (Fig. 6A). Also, an inverse association between VEGF-A_165_b levels and angiogenesis induced by serum (+IgG) from STEMI patients was also observed (r = −0.29, p = 0.03) (Fig. 6B).

Finally, we repeated the proliferation and migration assays in HCAEC using serum from STEMI patients and controls. We again observed that the treatment with an anti-VEGF-A_165_b neutralizing antibody significantly increased the proliferation (STEMI: 67.3±18.5% vs control: 36.4±18.9%, p < 0.01 (Fig. 6C) and the migration (STEMI: 81.2±48.6% vs control: 52.6±35.3%, p < 0.05 (Fig. 6D) of HCAEC cultured in serum from STEMI patients as compared with controls.

## Discussion

The endogenous VEGF-A isoform, VEGF-A_165_b, is known to inhibit endothelial proliferation and suppress tumor growth. However, little is currently known about the pathophysiology of VEGF-A_165_b in the setting of human myocardial infarction. To the best of our knowledge, ours is the first study to investigate VEGF-A_165_b expression and function in STEMI. The main findings are as follows: 1) systemic VEGF-A_165_b levels are elevated in STEMI patients; 2) elevated circulating VEGF-A_165_b levels are associated with a large infarct size and reduced LVEF in STEMI patients; 3) up-regulation of VEGF-A_165_b protein expression occurs in infarcted tissue; 4) VEGF-A_165_b blockade in serum from STEMI patients enhances angiogenesis, proliferation and migration of human coronary endothelial cells *ex vivo*.

Early reperfusion of the culprit coronary artery using primary PCI is the best available therapy for the management of STEMI patients. Nevertheless, despite its generalized use in western countries, severe microvascular damage can persist in a significant number of patients, resulting in larger infarctions, systolic deterioration, left ventricular remodeling, and worsening of patient outcomes^2, 29^. The development of neoangiogenesis within the myocardial infarct occurs as a response to ischemia to restore microvascular perfusion in the weeks and months following AMI, thus exerting salutary effects on infarct healing, systolic recovery and left ventricular dilation^1, 2, 30^. More knowledge on this issue is critical to better understand the pathophysiology of AMI and to develop new therapeutic opportunities beyond prompt coronary reperfusion, which can help to decrease the burden of AMI in terms of morbidity and mortality.

Angiogenesis is highly regulated and requires the orchestrated interaction of endothelial cells and surrounding cells mediated by growth factors and their receptors^3^. Among them, VEGF-A and its receptor VEGFR-2 have been shown to play major roles in many pathological scenarios that occur with angiogenesis, including AMI^8, 9^. VEGF-A expression is increased in the ischemic myocardium^6, 7^, and elevated serum VEGF-A levels have been reported in patients with STEMI^8, 9, 31^. However, VEGF-A can be differentially spliced to generate the inhibitory VEGF-A_165_b isoform, which has recently emerged as an important factor in several clinical ischemic states. Accordingly, elevated circulating VEGF-A_165_b levels have been reported in patients with peripheral artery disease^17–19^ or systemic sclerosis^20^. In the present study, we demonstrate that regulation of VEGF-A splicing takes place also in the setting of myocardial ischemia since VEGF-A_165_b expression was rapidly increased at early phases after AMI to reach a maximum level 24 h after reperfusion. Given the temporal characteristics of VEGF-A_165_b expression in peripheral blood, it is tempting to speculate that VEGF-A_165_b may contribute to the impaired neovascularization in the acute phase after myocardial ischemia.

CMR has become the gold standard non-invasive cardiac imaging technique to comprehensively characterize the structural consequences of AMI^32^. In particular, cine-CMR and delayed enhancement sequences allow for state-of-the-art quantification of two potent prognostic indices in post-STEMI patients, namely LVEF and infarct size, respectively^32, 33^. Interestingly, we show in our study that VEGF-A_165_b in peripheral blood from STEMI patients is associated with a more extensive infarct. Likewise, higher VEGF-A_165_b levels are associated with reduced LVEF. Thus, up-regulation of VEGF-A_165_b expression after STEMI is associated with severe LV deterioration.

Previous studies have demonstrated that VEGF-A_165_b is down-regulated in pathological angiogenic states such as cancer and diabetic retinopathy^14, 34, 35^. Conversely, up-regulation of VEGF-A_165_b is associated with reduced neovascularization in several diseases including obesity^36^, systemic sclerosis^20^, and peripheral artery disease^18, 37^. Along this line, we show an up-regulation of VEGF-A_165_b protein expression in the infarcted myocardial tissue of patients with an OMI. In accordance with previous reports^20^, VEGF-A_165_b was mainly localized to vascular endothelial cells. While we have no direct evidence that increased VEGF-A_165_b myocardial expression occurs early after MI and further research is needed, our results suggest that VEGF-A_165_b may be involved in neovessel formation late after MI.

The anti-angiogenic effects of VEGF-A_165_b have been well demonstrated in a large number of experimental and human studies^14, 18, 20, 22^. In this context, VEGF-A_165_b has been shown to reduce VEGF-A_165_-induced morphogenesis *in vitro* in dermal microvascular endothelial cells^20^ and in animal models^34, 38^. In line with these findings, we found that VEGF-A_165_b inhibited VEGF-A_165_-induced morphogenesis, proliferation and migration in human coronary endothelial cells. Additionally, we observed that VEGF-A_165_b blocked the phosphorylation (activation) of AKT induced by VEGF-A_165_ in HCAEC. AKT is involved in multiple signaling pathways in the regulation of angiogenesis^39^, and is reported to be relevant as a pro-survival signal in response to cardiac hypoxia and other stimuli such as VEGF-A^39^. Therefore, our present data suggest that the anti-angiogenic effect of VEGF-A_165_b in human coronary endothelial cells could be due, at least in part, to inhibition of VEGFR-2/AKT signalling.

Our *in vivo* observations prompted us to evaluate the potential functional effects of VEGF-A_165_b blockade for STEMI patients. We found that neutralization of VEGF-A_165_b activity in serum from STEMI patients significantly increased the angiogenic response. Considering that pro-angiogenic therapy based on VEGF-A administration in human clinical trials has been largely unsuccessful, overall, our data suggest that functional blockade of VEGF-A_165_b might constitute a new strategy to improve neovessel formation after AMI. In agreement with this, delivery of VEGF-A_165_b reduced revascularization of the ischemic hindlimb in a murine model of peripheral artery disease, whereas treatment with a neutralizing antibody of this isoform reversed impaired revascularization^18^.

Our data suggest that regulation of splicing of VEGF-A may also be important in the context of AMI in humans, implicating a novel mechanism of VEGF-A_165_b up-regulation that may relevant for the management of patients. We speculate that the high circulating levels of VEGF-A_165_b in STEMI patients might hamper the spontaneous tendency towards recovery of microvascular perfusion and, in turn, lead to a larger infarction with more severely depressed systolic function. Our *ex vivo* results point in this direction and strongly suggest that VEGF-A_165_b blockade is a promising therapeutic option to accelerate neoangiogenesis in the infarcted area. Nevertheless, further mechanistic studies are required to understand the molecular pathways involved in VEGF-A_165_b-associated cardiac angiogenesis. Additionally, the limitations of the present study include the small number of measured samples and its single-center design. Replication of these findings in other cohorts is needed as a next step in evaluating the importance of VEGF-A_165_b in mediating tissue reperfusion and its relationship with patient survival.

In conclusion, we provide evidence that endogenous VEGF-A_165_b is significantly elevated in STEMI patients. To our knowledge, this is the first report on a previously unexplored anti-angiogenic factor in the context of AMI that may be involved in the pathogenesis of STEMI in humans. Therefore, pharmacological modulation of VEGF-A_165_b expression and function might be a promising therapeutic strategy to accelerate angiogenesis after AMI.

## Methods

### Study population

The study conformed to the principles outlined in the Declaration of Helsinki for the use of human subjects. The study protocol was approved by the Ethics Committee of the University Clinic Hospital of Valencia. Written informed consent was obtained from all subjects. Reporting of the study conformed to the STROBE statement along with references to STROBE and the broader EQUATOR guidelines^40^.

Patients were considered to be included in the study group if they were admitted to our institution with a first STEMI defined following current definitions^41^, were treated with primary PCI within 12 hours of chest pain onset, and underwent CMR imaging at pre-discharge. We prospectively enrolled 81 consecutive patients from July 2013 to July 2014 with these characteristics.

Death (n = 1), re-infarction (n = 3), clinical instability (n = 2) during admission and any contraindication to CMR (n = 6) were exclusion criteria. Therefore, the final study group comprised 50 patients. The flow chart of patients in the study is presented in Fig. 1A.

We recruited a control group matched in age and sex with the study group, made up of 23 patients in whom the presence of any cardiac disease was ruled out by means of a thorough clinical history, physical exploration and echocardiographic study carried out by a clinical cardiologist.

### Baseline characteristics and serial blood samples

Baseline characteristics were prospectively registered in all cases. The PCI technique was chosen at the discretion of the interventional operator. Thrombolysis in Myocardial Infarction (TIMI) flow grade in the problem artery (before and after PCI) was analyzed^42^. Patients were managed both in-hospital and after discharge by a specific STEMI unit, and current recommendations^43^ were strictly followed. Further details on patients’ characteristics are shown in Table 1.

Blood samples were centrifuged at 2300 rpm for 15 min and serum was immediately stored at −80 °C. In the first 20 patients included in the study group, sequential blood sampling was obtained upon patient arrival (before primary PCI), and at 24 h, 96 h and 1 month after primary PCI. Sequential blood samples in the first 20 STEMI patients and 20 controls were used to define the temporal course of VEGF-A_165_b levels. VEGF-A_165_b peaked 24 h post-PCI. In patients 21–50, a single blood sample collected 24 h after primary PCI was obtained. The association between VEGF-A_165_b levels at 24 h post-PCI with infarct size (% of LV mass) and LVEF (%) as derived from CMR was determined in the 50 patients included in the final study group

### Cardiac Magnetic Resonance

CMR (1.5 T unit, Magnetom Sonata; Siemens, Erlangen, Germany) was performed 7±2 days after STEMI, in accordance with our laboratory protocol and current recommendations^1, 32^. All studies were performed by two cardiologists specialized in CMR with 15 years of experience. An experienced observer with 3 years of experience, blinded to all patient data, analyzed the CMR data offline using customized software (QMASS MR 6.1.5, Medis, Leiden, The Netherlands). CMR data were prospectively recorded and immediately included in the registry database. LVEF (%), LV end-diastolic volume index (mL/m^2^), LV end-systolic volume index (mL/m^2^), and LV mass index (g/m^2^) was calculated by manual planimetry of endocardial and epicardial borders in short-axis view cine images. Areas showing late gadolinium enhancement were visually revised and quantified by manual planimetry. Infarct size (% of LV mass) was assessed as the percentage of LV mass showing late gadolinium enhancement. Microvascular obstruction (% of LV mass) was quantified by manual planimetry and defined as the percentage of LV mass showing a lack of contrast uptake in the core of tissue showing late gadolinium enhancement. Myocardial edema was defined as areas of high T2 signal intensity. All short-axis view slices were separately analyzed and the presence of edema was visually revised, quantified by manual planimetry and expressed as percentage of LV mass. Myocardial salvage index was calculated by subtracting the mass of infarcted myocardium from myocardium showing edema and expressed as percentage of LV mass with myocardial edema.

Images were acquired by a phased-array body surface coil during breath-holds and were triggered by electrocardiography. Cine images were acquired in two-, three-, and four-chamber views, and in short-axis views using a steady-state free precession sequence (repetition time/echo time: 2.8/1.2 ms; flip angle: 58 degrees; matrix: 256 × 300; field of view: 320 × 270 mm; slice thickness: 7 mm).

Late gadolinium enhancement imaging was performed 10 to 15 minutes after administering 0.1 mmol/kg of gadolinium diethylenetriaminepentaacetic acid (Magnograf, Juste S.A.Q.F., Madrid, Spain) in the same locations as in cine images using a segmented inversion recovery steady-state free precession sequence (repetition time/echo time: 750/1.26 ms; flip angle: 45 degrees; matrix: 256 × 184; field of view: 340 × 235 mm; slice thickness: 7 mm). Inversion time was adjusted to nullify normal myocardium.

Black blood, T2-weighted short TI inversion recovery sequences in the same short-axis view as the cine sequences, all in mid-diastole, were carried out. A half-Fourier acquisition single-shot turbo spin echo multisection sequence was used (recovery time: two R-R intervals; echo time: 33 ms; inversion time: 170 ms; slice thickness: 8 mm; interslice interval: 2 mm; flip angle: 160 degrees; matrix: 256 × 151; bandwidth: 781 Hz/pixel). Additionally, a segmented turbo-spin echo sequence was obtained with one slice per breath-hold (recovery time: two R-R intervals; echo time: 100 ms; inversion time: 170 ms; slice thickness: 8 mm; interslice interval: 2 mm; flip angle: 180 degrees; matrix: 256 × 146; bandwidth: 235 Hz/pixel).

The inter-observer variability for the calculation of traditional CMR indexes used in the present study in our laboratory has been previously reported and is less than 5%^1^. In brief, inter-observer variability was calculated by comparing the differences between the measurements performed by two experienced operators in 30 CMR studies randomly sampled from the study group.

For the purposes of the present study, to explore the association of VEGF-A_165_b with left ventricular damage following STEMI, we focused on the two CMR indexes that traditionally have been more solidly associated with patients’ outcomes, namely LVEF and infarct size^7^.

### VEGF-A_165_b detection

Serum levels of VEGF-A_165_b and total VEGF-A were measured using commercially available ELISA kits (cat#MBS109074, My Biosource Inc. San Diego, CA and DY293B, R&D Systems, Minneapolis, MN, respectively), according to the manufacturers’ recommendations.

### Cell culture

Human coronary artery endothelial cells (HCAEC, Lonza, Barcelona, Spain) were cultured and maintained in human endothelial cell specific medium (EBM-2) supplemented with endothelial growth media (EGM-2) and 10% fetal bovine serum (FBS).

### Endothelial differentiation assay

Growth factor-reduced Matrigel (100 µL) (BD Biosciences, Madrid, Spain) was added to tissue culture 96-well plates and polymerized for 30 minutes at 37 °C as decribed^26^. HCAEC (30 × 10^3^ cells/well) were seeded into Matrigel in DMEM medium containing 2% FBS. Cells were treated with vehicle (PBS), human recombinant VEGF-A_165_ (30 ng/mL; cat#293VE, R&D Systems), VEGF-A_165_b (30 ng/mL; cat#3045VE, R&D Systems) or their combination (both at 30 ng/mL).

In additional experiments, HCAEC were seeded in Matrigel and incubated with diluted serum (10%) from STEMI patients (n = 50) and controls (n = 23). Additionally, samples were incubated in the presence of a mouse monoclonal anti-human VEGF-A_165_b blocking antibody (10 µg/mL, cat#MAB3045, R&D Systems) or an irrelevant isotype and concentration-matched IgG (10 µg/mL, R&D Systems) as previously described^20^. Phase contrast micrographs (Axio Observer A1, Carl Zeiss, NY) were recorded after 24 h and the number of tubes in 5 low-power (×100) random fields were counted. Scoring of tubulogenesis was performed by an investigator blinded to treatments.

### BrdU proliferation assay

Proliferation assays were performed by bromodeoxyuridine (BrdU) incorporation (50 μM) as previously described^27^. HCAEC were treated with vehicle (PBS), human recombinant VEGF-A_165_ (30 ng/mL), VEGF-A_165_b (30 ng/mL) or their combination (both at 30 ng/mL). In additional studies, HCAEC were seeded and incubated with diluted serum (10%) from STEMI patients and controls. Then, samples were incubated in the presence of a mouse monoclonal anti-human VEGF-A_165_b blocking antibody (10 µg/mL) or an irrelevant isotype and concentration-matched IgG (10 µg/mL). After 24 h, cells were fixed with 4% paraformaldehyde for 20 min, permeabilized with 1.0% Triton X-100 in PBS for 15 min, washed with PBS, and incubated with PBS containing 10% normal goat serum for 1 h at room temperature. BrdU-positive cells were detected by immunofluorescence with an anti-BrdU Alexa Fluor-488 antibody (Invitrogen, CA). 4′,6-diamidino-2-phenylindole (DAPI) was used for nuclear staining (total cell count). Cell proliferation was determined as the percentage of BrdU positive cells relative to total cell count.

### Wound-healing migration assay

Migratory activity was assessed by a standard wound-healing migration assay. HCAEC were plated on 12-well plates to 80% confluence. At time 0 of the migration assay, a wound was made in the center of the cell monolayer using a sterile tip and cells were incubated with the different treatments as described above for 24 h. Images were taken at time 0 h and 24 h. Cell migration was calculated as wound area closure of at least 5 points at time t = 24 relative to wound area at t = 0. Analysis was performed with ImageJ software.

### Western blotting

HCAEC were treated with human recombinant VEGF-A_165_ (30 ng/mL; cat# 293VE, R&D Systems), VEGF-A_165_b (30 ng/mL; cat#3045VE, R&D Systems) or their combination (both at 30 ng/mL) for 20 min. After treatments, cells were lysed and protein concentration was determined using the Bradford method^44^. Samples were denatured, subjected to SDS-PAGE using a 10% running gel, and transferred to a nitrocellulose membrane. Non-specific binding sites were blocked with 3% bovine serum albumin (BSA) in TBS solution and membranes were incubated overnight with rabbit polyclonal antibodies against human VEGFR2 (1:200 dilution, cat#ab39256, Abcam, Cambridge, UK), AKT(1/200 dilution, cat#4685, Cell Signaling Technology, Danvers, MA), and phospho-AKT (Ser473) (1/200 dilution, cat#4085, Cell Signaling Technology). Membranes were subsequently washed, incubated for one additional hour with the secondary HRP-linked anti-rabbit antibody (1:2000 dilution, cat#0448, Dako, Glostrup, Denmark) and developed using an ECL procedure (GE Healthcare, Madrid, Spain). Signals were recorded using a luminescent analyzer (FujiFilm Image Reader LAS 4000, Fuji, Tokyo, Japan).

### VEGF-A_165_b detection in myocardial tissue samples

Postmortem myocardial tissue was obtained from the autopsies of four patients with an OMI (samples were obtained more than 6 months after AMI occurrence). A previous history of AMI had been previously documented in all patients, and autopsies unequivocally revealed a fibrotic scar characteristic of chronic infarct in all cases. Additionally, myocardial samples of the autopsies of four patients with no evidence of myocardial infarction or other structural cardiac abnormalities were used as controls; a previous history of ischemic or any other cardiac diseases had been ruled out both in the clinical records and in the autopsy analyses. The clinical data and autopsy results of patients are described in Table 2. Heart tissue samples were fixed in 10% formalin at 4 °C for 24 h, embedded in paraffin, sectioned (5 μm) and mounted on double gelatin-coated glass slides. Collagen deposition was detected by staining with picrosirius red. Briefly, dehydrated sections were incubated in 0.2% phosphomolydbic acid and then stained with 1% Direct Red 80 (Sigma-Aldrich, Madrid, Spain) in saturated picric acid and washed in 0.01 N hydrochloric acid as previously described^45^. For VEGF-A_165_b detection, sections were treated with 0.25% trypsin in 9 mmol/L CaCl_2_/50 mmol/L Tris-HCl, pH 7.8, for 30 minutes at room temperature for antigen retrieval. After blockade with goat serum (3%), samples were incubated overnight (4 °C) with the following primary antibodies diluted in 0.1% PBS/BSA: mouse anti-human VEGF-A_165_b (5 μg/ml, cat# MAB3045, R&D Systems), or control IgG (5 μg/ml, cat# MAB002, R&D Systems). Specific labeling was detected with a biotin-conjugated goat anti-mouse secondary antibody (1:500 dilution, Dako) or with Alexa Fluor 594 goat anti-mouse secondary antibody (1:1000 dilution, cat# A11005, ThermoFisher Scientific). For double immunofluorescence analysis, VEGF-A_165_b was detected with a mouse anti-human VEGF-A_165_b antibody (5 μg/ml, cat# MAB3045, R&D Systems), CD31 with a rabbit polyclonal anti-human-CD31 (1:50 dilution, cat# ab32457, Abcam) and VEGFR2 with a rabbit polyclonal anti human-VEGFR2 (1:20 dilution, cat# ab39638, Abcam). Specific labeling was detected with an Alexa Fluor 594 goat anti-mouse secondary antibody (1:1000 dilution, cat# A11005, ThermoFisher Scientific) or an Alexa Fluor 488 goat anti-rabbit secondary antibody (1:1000 dilution, cat# A11034, ThermoFisher Scientific). Fields from each infarct or control sections were captured, digitized and then analyzed with Image-Pro Plus analysis software (Media Cybernetics, Inc., Rockville, MD). Scoring was performed blinded on coded slides.

### Statistical analysis

We assessed normality of distribution with the Kolmogorov-Smirnov test. Continuous normally distributed data were expressed as the mean ± the standard deviation of the mean, whereas non-parametric data were expressed as the median and the interquartile range. For comparisons of two groups, Student’s t test was used for data that passed both normality (Kolmogorov-Smirnov test) and equality of variance (Levene’s test); otherwise, the non-parametric Mann Whitney U test was used. For comparisons among multiple groups, one-way analysis of variance (ANOVA) followed by *post hoc* analysis (Bonferroni test) was used in data that passed both normality and equality of variance; otherwise, the non-parametric Kruskal–Wallis test followed by Dunn’s *post hoc* analysis was used. The Spearman correlation test was used to analyze the correlation between VEGF-A_165_b levels and different clinical variables of the patients. Group percentages were compared using the Chi-square test or Fisher’s exact test where appropriate. Data were analyzed using GraphPad software (GraphPad Prism 4, Inc, La Jolla, CA). Data were considered statistically significant at p<0.05.

## Electronic supplementary material

Supplementary File
