# Supplementary material for: Dynamics and implications of circulating anti-angiogenic VEGF-A165b isoform in patients with ST-elevation myocardial infarction
Source: Sci Rep. 2017 Aug 30;7:9962. doi: 10.1038/s41598-017-10505-9 (PMC5577291; doi:10.1038/s41598-017-10505-9)
Supplement: Supplementary file 1 — Supplementary File [file 41598_2017_10505_MOESM1_ESM.pdf]

Dynamics and implications of circulating anti-angiogenic VEGF-A_{165}b isoform in patients with ST-elevation myocardial infarction

Luisa Hueso [1#], Cesar Rios-Navarro [1,2#], Amparo Ruiz Sauri [1,3], Francisco Javier Chorro [1,2,4,5], Julio Nunez[1,2,4], Maria Jesus Sanz [1,6], Vicente Bodi [1,2,4,5]* & Laura Piqueras [1]*

[1]Institute of Health Research-INCLIVA, Valencia, Spain
[2]Cardiology Department, Hospital Clinico Universitario, Valencia, Spain.
[3]Pathology Department, Faculty of Medicine, University of Valencia, Valencia, Spain
[4]Medicine Department, Faculty of Medicine, University of Valencia, Valencia, Spain
[5]Centro de Investigación Biomédica en Red - Cardiovascular (CIBER-CV)
[6]Pharmacology Department, Faculty of Medicine, University of Valencia, Valencia, Spain
[#]Both authors contributed equally to this work

***Authors for correspondence:** Dr. Laura Piqueras; piqueras_lau@gva.es and Dr. Vicente Bodi; vicente.bodi@uv.es

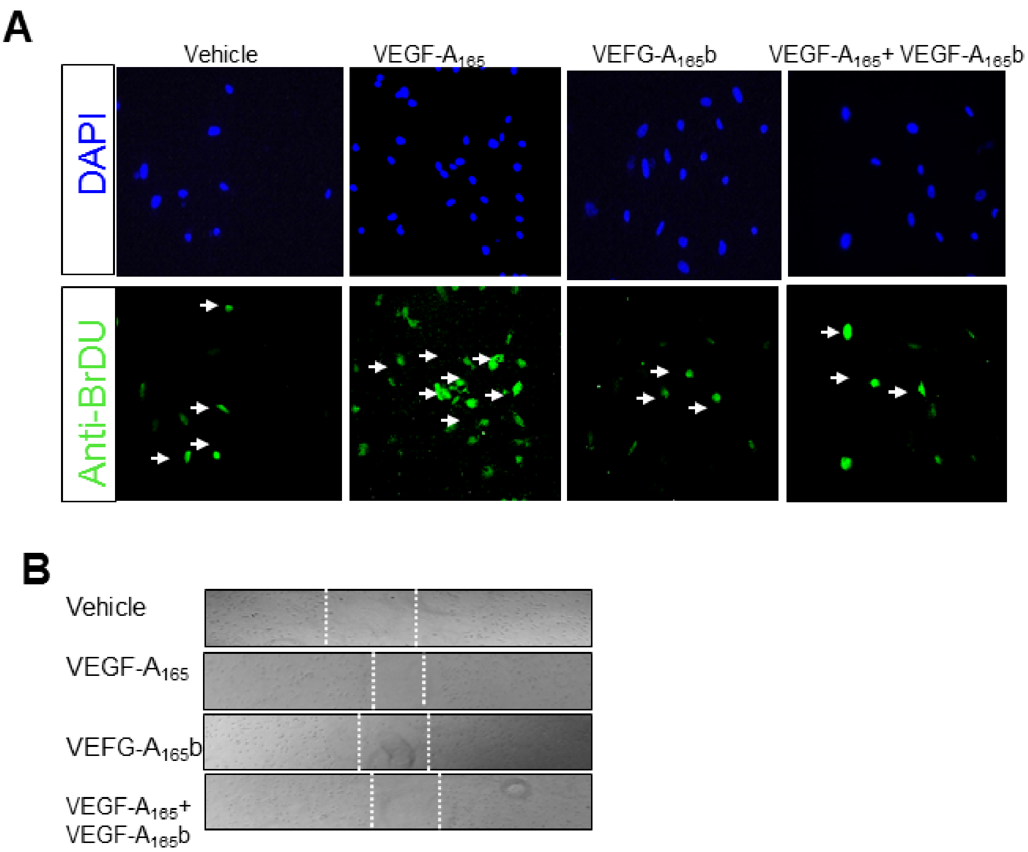

Supp Figure S1. A, VEGF-A_{165}b inhibits VEGF-A_{165}-induced proliferation and migration of human coronary artery endothelial cells (HCAEC). HCAEC were treated with vehicle (PBS), human recombinant VEGF-A_{165} (30 ng/mL), recombinant VEGF-A_{165}b (30 ng/mL) or their combination **A,** Representative images showing proliferating cells analyzed by BrdU incorporation and detected by immunofluorescence. Arrows point to cell nuclei**. B,** Representative images of wound-healing migration assay with the different treatments are shown.

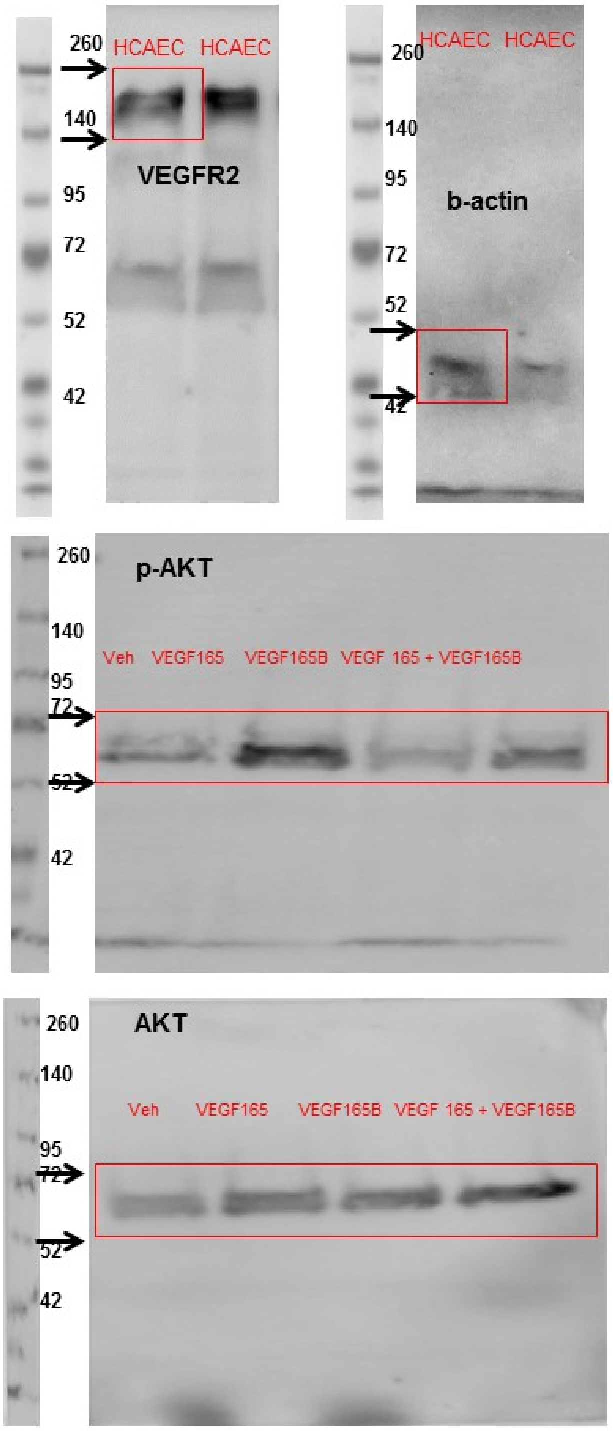

Full length immunoblots relating to Figure 5. VEGFR2, b-actin, p-AKT and AKT protein are showed. Signals were recorded using a luminescent analyzer (FujiFilm Image Reader LAS 4000). Broad range protein ladder was from Thermo scientific (cat. number 26634)
